# Body Temperature Detection of Group-Housed Pigs Based on the Pairing of Left and Right Ear Roots in Thermal Images

**DOI:** 10.3390/ani15050642

**Published:** 2025-02-22

**Authors:** Rong Xiang, Yi Zhang, Hongjian Lin, Yingchun Fu, Xiuqin Rao, Jinming Pan, Chenghao Pan

**Affiliations:** 1 College of Quality and Standardization, China Jiliang University, Hangzhou 310018, China; yizhang_30@163.com (Y.Z.); chpan@cjlu.edu.cn (C.P.); 2 College of Biosystems Engineering and Food Science, Zhejiang University, Hangzhou 310058, China; linhongjian@zju.edu.cn (H.L.); ycfu@zju.edu.cn (Y.F.); xqrao@zju.edu.cn (X.R.); panhouse@zju.edu.cn (J.P.)

**Keywords:** group-housed pigs, ear root, body temperature, thermal images, YOLO

## Abstract

Body temperature is a critical indicator of pig health. This study proposes a non-contact method for detecting body temperature in group-housed pigs by extracting temperature data from thermal images of ear roots. Thermal images in the drinking trough area were captured using a thermal camera, with real-time data transmitted to a monitoring room via optical fibers. The YOLO v11m-OBB model was utilized to detect the ear root areas with oriented bounding boxes, while a novel algorithm, the two-stage left and right ear root pairing algorithm (YOLO TEPA-OBB), paired the ear roots of individual pigs using center distance clustering and angular relationships in a polar coordinate system. The maximum temperature of the ear roots was extracted to represent the body temperature. Experimental results based on 749 ear roots show that the YOLO TEPA-OBB achieves 98.7% precision, 98.4% recall, and 98.7% mean average precision (*mAP*) in detecting ear roots, with an ear root pairing accuracy of 98.1%. The Pearson correlation coefficient (*r*) between predicted and reference temperatures is 0.989, with a mean bias of 0.014 °C and a standard deviation of 0.103 °C. This research facilitates real-time body temperature monitoring and precise health management for group-housed pigs.

## Simple Summary

Monitoring the body temperature of pigs is crucial for ensuring their health, especially in group-housed pigs. This study focuses on a new method for measuring pigs’ body temperature without physical contact, using thermal images captured by a thermal camera. The method involves detecting the temperature at the roots of pigs’ ears, which is a reliable indicator of their overall body temperature. To accurately identify and pair the left and right ear roots, a novel algorithm was developed. This system allows for precise temperature readings even when pigs are in different positions and postures. The results show that the system is capable of detecting ear roots with high accuracy and the temperature readings are highly reliable. The findings of this study can be used to improve the real-time monitoring of pig health, making it easier to manage their well-being and prevent diseases, ultimately benefiting both animal welfare and farm productivity.

## 1. Introduction

Infectious diseases, such as swine fever, present a significant threat to the pig farming industry. If these diseases are not detected promptly, they can result in significant economic losses. Therefore, real-time monitoring of pig health is essential for early detection and effective disease management [1,2]. Body temperature is a crucial physiological indicator of pigs’ health status. Real-time, accurate monitoring of pig body temperature not only facilitates the early prevention of diseases like swine fever but also enhances overall farming quality and efficiency. Research on accurate and efficient methods for detecting pig body temperature is thus of great importance [3,4,5,6].

Pig body temperature is usually measured by mercury thermometers, electronic thermometers, and implantable sensors [7,8]. Traditional methods rely on manual rectal temperature measurements using thermometers. While accurate, this method is time-consuming, labor-intensive, and stressful for the pigs, increasing the risk of cross-contamination. As such, it fails to meet the demands of modern, intensive, and automated farming systems [9]. Implantable sensors measure temperature by converting thermal signals into electrical signals. These sensors are small and can be implanted or embedded in the pig’s body surface [10]. Hentzen et al. invented a capsule-type wireless sensor that can be implanted in the neck of pigs, providing measurement results consistent with manual rectal temperature readings [11]. Zhang Ziyun et al. used a new type of electronic chip to measure the subcutaneous body temperature of different breeds of gilts [12]. While implantable sensors offer accurate temperature data, they cause considerable harm to the pigs, compromise animal welfare, and incur high costs, as each pig requires an individual sensor implant.

In recent years, non-contact temperature measurement methods using thermal infrared equipment have gained prominence and have been widely used for livestock body temperature monitoring and disease diagnosis [13,14,15,16]. Infrared thermometry is classified into point-source and area-source measurements. Gao Libo et al. measured the body temperature of 1000 pigs at a slaughterhouse using a mercury thermometer, electronic thermometer, and infrared thermometer. By comparing the rectal temperature measured with the mercury thermometer and the surface temperature of the ear root measured with the infrared thermometer, they found a consistency rate of 93% for detecting pigs with high temperatures, thus validating the feasibility of infrared thermometry on pig ear roots [17]. Soerensen and Pedersen evaluated the application of infrared thermometry for measuring the temperature of breeding pigs and concluded that it is a potentially efficient temperature measurement method [18]. Point-source infrared thermometry has developed rapidly due to its portability and ease of use. However, it requires manual measurement of each pig, making it labor-intensive. Moreover, since the measurement points are chosen randomly, the results can suffer from large random errors. In contrast, area-source infrared thermometry uses infrared thermal cameras to convert thermal radiation into electrical signals, which are then processed by designed algorithms to perform body temperature measurements. This method is based on thermal field analysis, enabling full-field temperature detection and overcoming the random error limitations of random measurement points associated with handheld thermometers [19,20].

Area-source infrared thermometry often integrates deep learning and computer vision techniques. Thermal images of pigs are captured using infrared thermal cameras, and deep learning models are trained to identify the regions of interest for temperature measurement, from which temperature values are extracted to represent the body temperature of pigs [21,22,23]. Feng Yankun et al. identified the optimal ear root measurement area for pigs in feeding stalls based on their head movement trajectories, using object recognition algorithms to achieve accurate body temperature measurement. However, this method requires pigs to pass through a narrow channel sequentially, limiting its efficiency [24]. Xiao Deqin et al. developed an inspection device equipped with a thermal image-based ear detection algorithm, extracting the maximum and average temperature values from the ear region to represent pig body temperature. This method requires sows to be confined in stalls, which makes it unsuitable for free-range environments [25]. Xie Qiuju et al. employed a handheld infrared thermal camera to capture thermal images of pigs’ foreheads and ear roots, using object detection algorithms to extract individual pig forehead and ear root temperatures. This method requires manual operation of the thermal camera, making it inefficient and capable of detecting the ear root temperature of only a single pig at a time [26]. Liu Gang et al. used an improved YOLOv7 object recognition algorithm to locate the head region of group-housed pigs in thermal images, extracting the maximum temperature from the head region to measure body temperature for group-housed pigs [27]. Current studies indicate that ear root temperature is significantly correlated with the true body temperature of pigs. For example, Tabuaciri et al. chose the ear root, ear tip, and back as measurement sites for piglets and found that the ear root surface temperature had the highest correlation with rectal temperature (*R* = 0.85) [28]. Consequently, area-source infrared thermometry often uses the ear root region as the region of interest for temperature measurement [29]. In group-housed environments, pigs often cluster together, with the ear roots of individual pigs being close to those of other pigs, sometimes even instances of adhesion or overlapping, making the identification of individual ear root areas of pigs more challenging. Additionally, temperature measurements based on a single ear root can be influenced by the pig’s posture, introducing considerable randomness.

This paper proposes a method for individual body temperature detection of group-housed pigs based on the detection, recognition, and matching of left and right ear roots in thermal images. The main objectives of this study are (1) to identify the ear root regions of group-housed pigs from thermal images, (2) to pair the left and right ear roots of individual pigs, and (3) to detect the body temperature of individual pigs in the group.

## 2. Materials and Methods

### 2.1. Image Dataset

#### 2.1.1. Thermal Image Collection

**Figure 1:**
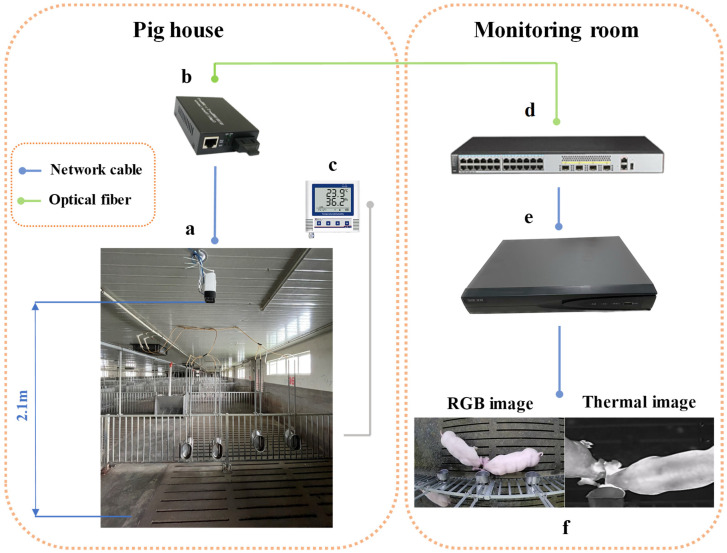
Thermal image collection system for pigs. **a**. Thermal camera; **b**. fiber-optic transceiver; **c**. temperature and humidity logger; **d**. switch; **e**. DVR; **f**. captured images.

The thermal images were captured in a pig breeding room at the Technology Ranch of the Institute of Animal Husbandry, Zhejiang Academy of Agricultural Sciences. The subjects of image capture were White Landrace pigs in the fattening phase. In accordance with animal welfare principles, the breeding room housed 20 pigs, with an average activity space of 1.2 square meters per pig [30]. Images were captured at the end of the nursery phase, when the pigs transitioned into the fattening phase, and continued until the pigs were sold. The collection occurred from January to June 2024, with continuous 24-h video data collection over a period of 143 days. The image acquisition system is shown in Figure 1. The image acquisition device was the Hikvision thermal camera DS-2TD2636B-13/P, configured in a professional temperature measurement mode with an emissivity of 0.97 [31]. The thermal camera was installed at a height of 2.2 m above the drinking trough in the pigpen, at a distance of 2.1 m, capturing images from a top-down perspective to ensure that all pigs were within the camera’s field of view over a given period. Additionally, as the pigs maintain a head-down standing posture while drinking, their ear roots are fully exposed, facilitating subsequent ear root detection and body temperature measurement. The thermal infrared video data collected by the camera were transmitted via Ethernet to a fiber-optic transceiver, which connected to a switch in the monitoring room. The data were then stored in a Hikvision digital video recorder (DVR). The pigpen was equipped with temperature and humidity loggers to record the relative temperature and humidity parameters within the pen. Since the data collection method was non-contact, the pigs were not subjected to any stress or harm during the process, preventing negative effects such as stress. After the equipment was installed, the pigpen was disinfected, sterilized, and cleaned before the pigs were introduced, ensuring minimal human-pig contact to prevent disease transmission.

#### 2.1.2. Dataset Construction

Images were extracted and saved from thermal imaging videos during the period when pigs were more active, from 8 a.m. to 12 p.m. The images contain varying numbers and poses of pigs, with a resolution of 384 pixels × 288 pixels, resulting in a total of 1500 thermal images, which were compiled into a dataset. Since every pig would present at the drinking trough to drink over a period of time and stay in the thermal camera field of view, all 20 pigs were included in the construction of the dataset. Furthermore, to ensure that the bounding boxes fit the ear root region more tightly, oriented bounding boxes were used for annotation. Using the open-source annotation tool roLabelImg for visualization, all pig ear root areas in the dataset images were annotated, with manual adjustments made to the rotation angle and size of the oriented bounding boxes to fit the elongated pig ear root areas, thereby improving the localization accuracy of the ear root areas. The labels for the left and right ear roots were set as “Left ear root” and “Right ear root”, respectively. During annotation, the oriented bounding box was aligned parallel to the ear root line, covering both the ear root line and its highlighted regions on the left and right. After annotation, XML-formatted labels were generated. In accordance with the YOLO dataset format, these XML labels were converted into a TXT format recognizable by the model. Each label’s specific information was represented as [*class_index*, $x_{1}$, $y_{1}$, $x_{2}$, $y_{2}$, $x_{3}$, $y_{3}$, $x_{4}$, $y_{4}$], representing the class index value and the coordinates of the four vertices of the oriented bounding box. Finally, the annotated dataset was split into training, validation, and test sets in a 7:1:2 ratio to build the YOLO-OBB format dataset.

#### 2.1.3. Experimental Platform and Parameter Settings

The software and hardware configurations for the experimental platform are shown in Table 1.

The training parameters were set as follows: the number of epochs was 400, the batch size was 16, and the initial learning rate was 0.01. To improve model training efficiency, a transfer learning strategy was employed, loading the pre-trained weights “yolo11m-obb.pt” provided by Ultralytics YOLO. Additionally, to make better use of computational resources and avoid overfitting, an early stopping strategy was adopted. Training was terminated early when there was no improvement in the validation loss for 50 consecutive epochs, indicating convergence of the model.

### 2.2. Method

The real-time processing workflow of the system is as follows: First, the video data captured by the thermal camera were transmitted in real-time to the server hosting the algorithm model via a network. Second, upon receiving each frame of the video, the server employed the trained YOLO v11m-OBB model to detect and locate the ear root regions and subsequently applied the TEPA algorithm for pairing and temperature extraction of ear root regions. This process was performed in real-time for each frame to ensure the immediate output of the pig’s body temperature.

**Figure 2:**
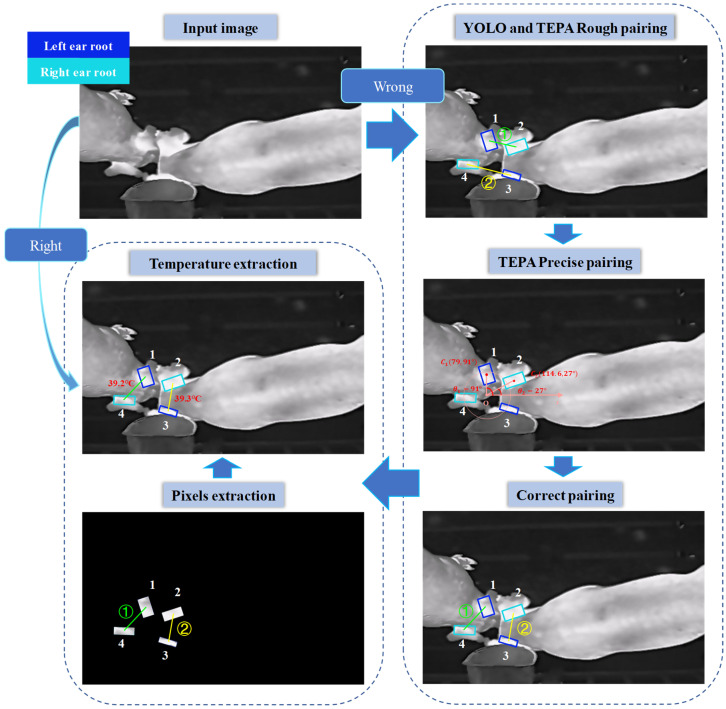
Flowchart for individual temperature detection of group-housed pigs. (‘1–4’ refers to the ear root bounding boxes, and “➀–➁” refers to the number of ear root pairs.).

The flowchart of the individual pig body temperature detection method in a group environment is shown in Figure 2. The oriented bounding box (OBB) target detection model, YOLO v11m-OBB, was used for ear root region detection and localization. The left and right ear root pairing algorithm, based on center distance clustering of predicted bounding boxes and the polar coordinate system, was used to pair the individual pig’s left and right ear roots. The temperature detection method extracted the maximum temperature from the predicted bounding boxes of the left and right ear roots to represent the body temperature of the pig.

### 2.3. YOLO v11m-OBB Ear Roots Detection Model

**Figure 3:**
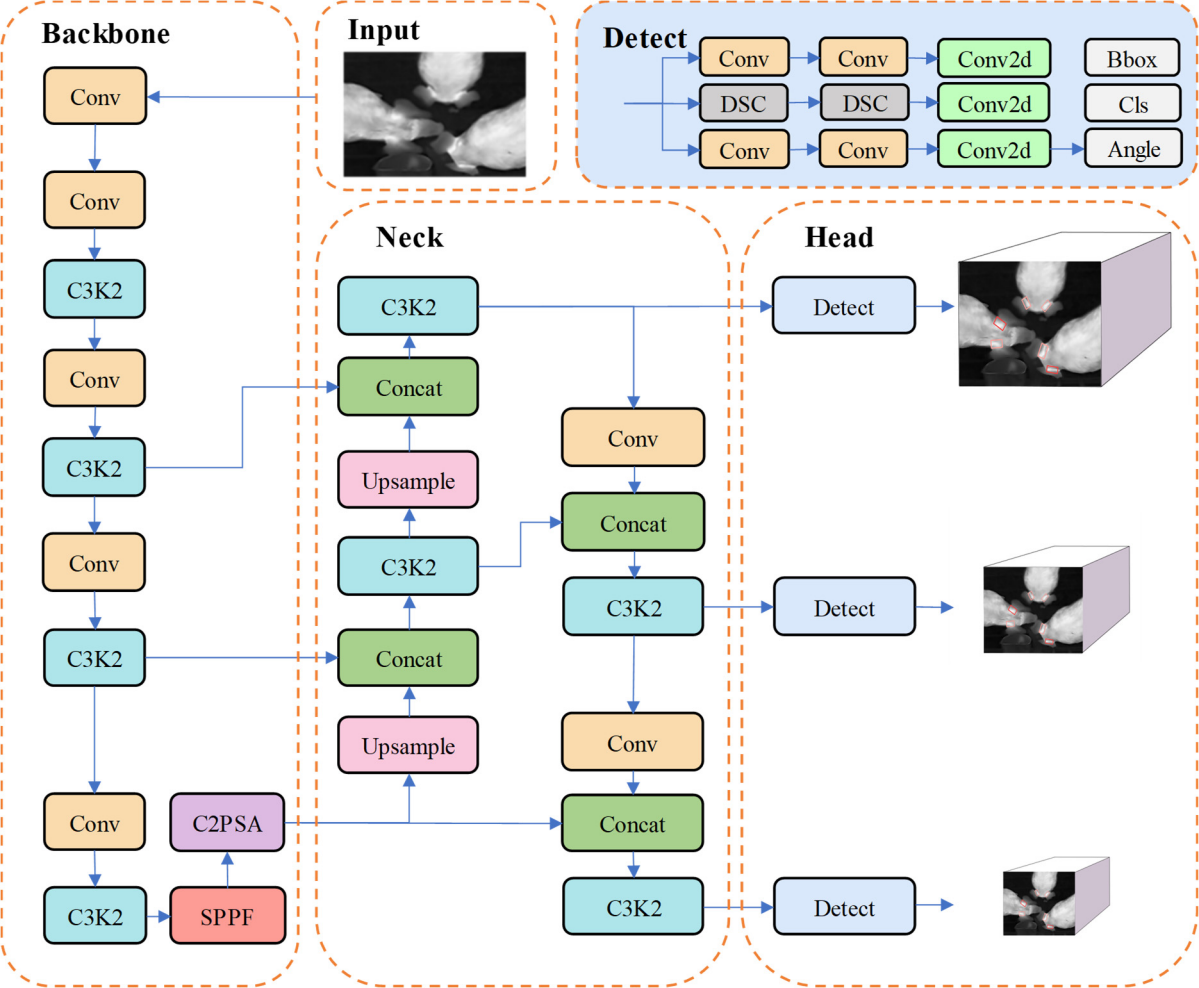
YOLO v11m-OBB network architecture.

The YOLO v11m-OBB (oriented bounding box) model is designed for directional target detection tasks and offers advantages such as high accuracy, real-time performance, and efficiency. Thermal images revealed that the ear root area of the pig exhibited the highest temperature, and the temperature difference compared with the auricular region was significant, making the ear root area a critical region for body temperature detection. Consequently, ear root regions are detected and located to enhance the accuracy of body temperature detection. The oriented bounding box (OBB) detection model can effectively fit the ear root regions with different orientations and reduce interference from background pixels, thereby enhancing the accuracy of detection. It integrates multi-scale feature representation capabilities, adds an attention mechanism, and incorporates the OBB mechanism to enhance adaptability to object poses and directions. The YOLO v11m-OBB model consists of the Input, Backbone, Neck, and Head layers, as shown in Figure 3.

The Input layer is responsible for receiving and preprocessing the dataset images. The Backbone is composed of Conv modules, C3K2 modules, SPPF modules, and C2PSA attention modules, aiming to extract multi-scale, deep-level features from the input images. The addition of the PSA attention mechanism makes the model more sensitive to feature locations and class information. The Neck layer consists of Conv modules, C3K2 modules, Concat modules, and Upsample modules, employing PAN-FPN to concatenate feature maps along both bottom-up and top-down paths, thereby achieving multi-scale feature fusion and enhancing the expressive ability of features at different scales. The Head layer consists of detection heads, classification heads, and angle heads. The classification head in v11 uses depthwise separable convolutions instead of conventional convolutions to reduce parameters and computational complexity. The angle head, unique to the OBB model, is used to predict the rotation angle of objects in the feature maps. This enables the identification of pig ear root regions from different angles and the generation of corresponding oriented bounding boxes [32,33].

### 2.4. Two-Stage Left and Right Ear Root Pairing Algorithm (TEPA)

Due to variations in the distance and angle between the ears and the thermal imaging camera, the pig’s posture, head orientation, the influence of hair sparseness, and the presence of stains at ear roots, body temperature detection based on a single ear root will result in random errors. Using paired double ear roots as the temperature measurement regions for an individual pig can effectively mitigate the effects of the above random errors. The YOLO v11m-OBB model predicts the regions of the left and right ear roots in an end-to-end manner, producing independent bounding boxes for each ear root. Therefore, it is necessary to pair the prediction bounding boxes of left and right ear roots belonging to the same pig. In group settings, pigs frequently cluster near drinking troughs, resulting in closely connected or occluded ear roots of different pigs, which can lead to incorrect pairings of the left and right ear roots. To address this challenge, a two-stage ear root pairing algorithm is proposed. In the first stage, rough pairing is conducted by minimizing the center distance between the predicted bounding boxes of the left and right ear roots. In the second stage, precise pairing is achieved by analyzing the angular relationships of the bounding boxes in the polar coordinate system.

#### 2.4.1. Rough Pairing Algorithm Based on the Minimum Center Distance of Left and Right Ear Root Predicted Bounding Boxes

During rough matching, a threshold range is set for the center distance between the predicted left and right ear root bounding boxes of the pigs in the thermal image, and the left and right ear root regions within this range are identified as belonging to the same pig.

**Figure 4:**
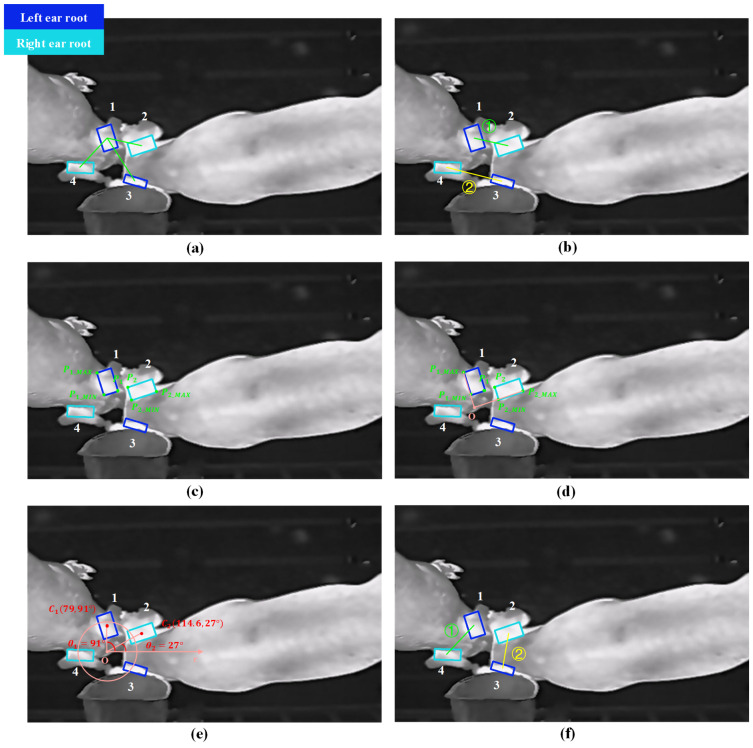
TEPA algorithm workflow. (**a**) Center distance calculation; (**b**) rough pairing; (**c**) calculation of base and outermost points; (**d**) extraction of outermost lines and intersection points; (**e**) left and right ear classification; (**f**) re-pairing. (‘1–4’ refers to the ear root bounding boxes, and “①–②” refers to the number of ear root pairs.).

Based on this threshold range, a rough pairing algorithm is proposed, utilizing the minimum center distance between the predicted left and right ear root bounding boxes. The algorithm workflow is illustrated in Figure 4a,b. First, the center coordinates of all predicted bounding boxes in the image are calculated. Then, the distances between the center points of every pair of bounding boxes are computed. The two bounding boxes with the smallest center distance are identified as the predicted left and right ear root regions for a single pig and are subsequently removed from the unpaired list. This process is iteratively repeated until all bounding boxes are paired. To ensure the accuracy of the pairing, the center distance range is restricted to between 80 and 160 pixels. Bounding boxes with center distances outside this range are considered unrelated to the same pig.

#### 2.4.2. Precise Pairing Algorithm for Left and Right Ear Roots Based on Polar Coordinate System

After rough pairing, incorrect pairings may still occur due to the proximity or adhesion of ear regions in a group-rearing environment. Therefore, this study further proposes a precise left and right ear root pairing algorithm based on the polar coordinate system.

The ear root regions of pigs typically form a “八” shape (resembling the Chinese character for “eight”), with the narrow end pointing toward the pig’s head and the wide end toward the pig’s body. Despite variations in the pigs’ standing or lying postures in the images, the relative positions and orientations of the left and right ear roots remain consistent with respect to the head direction. A precise left and right ear root pairing algorithm, based on the polar coordinate system, is employed to automatically determine the direction of the pig’s head and establish the polar coordinate system as a reference. By calculating the angles of the centers of the predicted left and right ear root bounding boxes within this coordinate system, the algorithm accurately classifies the left and right ear roots. If the results differ from the YOLO v11m-OBB’s initial predictions, it suggests a rough pairing error. In such cases, the rough pairing results are discarded, and the corresponding ear root bounding boxes are re-paired with other nearby predicted bounding boxes.

In Figure 4, due to two pigs’ heads touching, the minimum center distance between predicted bounding boxes 1 and 2 resulted in an incorrect rough pairing of the left and right ear root bounding boxes for the same pig.

The flow of the precise left and right ear root pairing algorithm based on the polar coordinate system is as follows:

First, the coordinates of the vertices of the ear root predicted bounding boxes returned by the YOLO v11m-OBB model are used to calculate the straight-line equations of the outermost lines of the left and right ear root predicted bounding boxes obtained through rough pairing, which help determine the direction of the pig’s head. The specific calculation method is as follows: the algorithm calculates the nearest pair of vertices between the four vertices of the ear root predicted bounding box 1 and the four vertices of the paired ear root predicted bounding box 2, designating them as base points $P_{1}$ and $P_{2}$. Then, it calculates the vertices $P_{1\_MIN}$ and $P_{2\_MIN}$, which are closest to the base points within each ear root predicted bounding box, and the vertices $P_{1\_MAX}$ and $P_{2\_MAX}$ that are furthest from the base points, as shown in Figure 4c. The lines connecting $P_{1\_MIN}$ and $P_{1\_MAX}$, and $P_{2\_MIN}$ and $P_{2\_MAX}$ represent the outermost lines of the left and right ear root predicted bounding boxes, respectively, as depicted in Figure 4d. Next, the intersection point *O* of the two outermost lines is calculated. The equations of the two outermost lines are represented by the point-slope formula, as shown in Equation (1):

(1) $$y-y_{1}=\frac{y_{2}-y_{1}}{x_{2}-x_{1}}(x-x_{1})$$

Using the intersection point *O* as the origin, a polar coordinate system is established, and the coordinates of the center points of the two predicted bounding boxes are calculated: $C_{1}(79,91{}^{\circ})$, $C_{2}(114.6,27{}^{\circ})$. The angle difference θ between these two points is calculated using Equation (2), as shown in Figure 4e:

(2) $$\theta =\left|\theta_{1}-\theta_{2}\right|=\left|91{}^{\circ}-27{}^{\circ}\right|=64{}^{\circ}$$

Based on the value of θ, the classification of the left and right ear root predicted bounding boxes are identified.

If θ<180°, the predicted bounding box with the smaller angle is classified as the left ear root, and the other one as the right ear root.

If θ>180°, the predicted bounding box with the larger angle is classified as the left ear root, and the other one as the right ear root.

For ear root predicted bounding boxes whose classification results differ from those of the YOLO v11m-OBB model, the polar coordinate system’s classification results are used to re-pair the left and right ear root predicted bounding boxes based on the minimum center distance. This requires discarding the initially incorrect rough pairing. As shown in Figure 4f, the rough pairing results of two left and right ear root pairs are incorrect. Since the angle of $C_{2}$ is smaller, it is reclassified as the left ear root, while $C_{1}$, with a larger angle, is reclassified as the right ear root, which contradicts the initial classification, indicating a pairing error. The predicted bounding boxes of the two ear roots are then re-paired based on the minimum center distance, thus correcting the error from the rough matching. This method is applied to all the rough-paired left and right ear root predicted bounding boxes, with the objective of optimizing the pairing algorithm’s performance.

### 2.5. Temperature Extraction from Ear Root Regions

Based on the strong correlation between the temperature of pig ear roots and their actual body temperature, the ear root regions are selected as the region of interest (ROI) for body temperature detection in pigs.

#### 2.5.1. Temperature Extraction Using Grayscale Linear Interpolation

**Figure 5:**
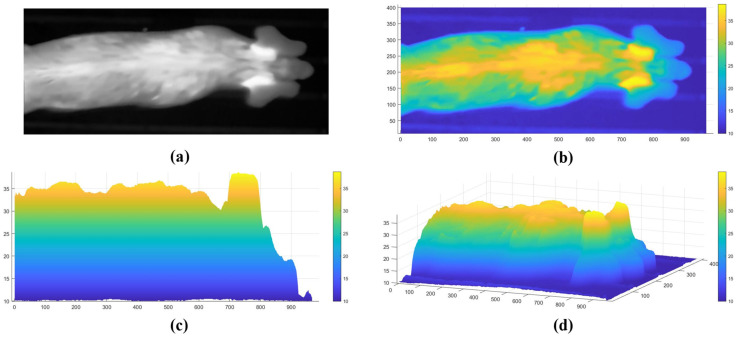
Visualization of pig body temperature distribution. (**a**) Original thermal image; (**b**) 3D body temperature distribution (top view); (**c**) 3D body temperature distribution (side view); (**d**) 3D body temperature distribution (3D view).

A linear interpolation method is employed, as shown in Equation (3), to map the pixel grayscale values of the left and right ear root predicted bounding boxes (ranging from 0 to 255) to the minimum and maximum temperature values recorded in the thermal image, thus converting the grayscale values into temperature values. The resulting visualized body temperature distribution of the pigs is shown in Figure 5. It is evident that the ear root regions have the highest temperature on the pig’s body surface.

(3) $$T=\frac{T_{max}-T_{min}}{255}\times P+T_{min}$$

where *T* is the predicted body temperature, *P* is the pixel grayscale value, and $T_{max}$ and $T_{min}$ are the maximum and minimum temperature values in the thermal image.

#### 2.5.2. Ear Root Temperature Representation

A statistical analysis was conducted on the reference values and predicted ear root temperatures for 100 pigs.

**Figure 6:**
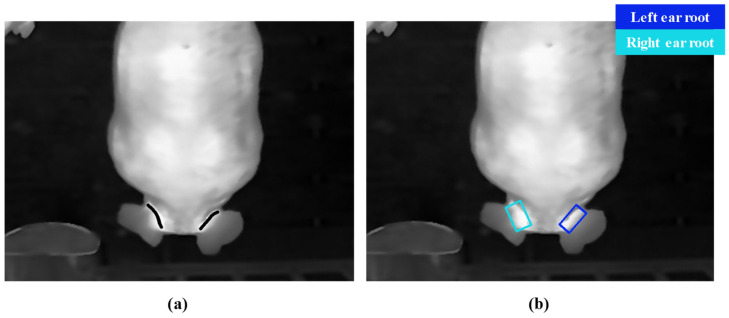
Extraction of reference and predicted values. (**a**) Reference value extraction (ear root line in bold black); (**b**) predicted value extraction.

The method for obtaining reference values is as follows: The central region of the pig’s ear root was manually delineated in the area with the highest brightness in the thermal image, with a width of 5 pixels. After the measurement of the width of the high-intensity region in the ear root regions, the modal value (i.e., a width of 5 pixels) of the ear root width was used to draw ear root lines. The maximum temperature value within this central region was taken as the reference value. The maximum temperature values were extracted from the predicted bounding boxes for each pig’s left ear root, right ear root, and both ear roots, forming three sets of predicted body temperature values for the pigs, as shown in Figure 6.

A correlation and consistency analysis was conducted between the predicted body temperature values and the reference values to evaluate the accuracy of the temperature predictions. The correlation analysis method involves performing linear fitting of the predicted and reference values, using the Pearson correlation coefficient (*r*) to evaluate the correlation between the predicted and reference values. The consistency analysis method applies measurement system analysis (MSA) bias analysis to evaluate the consistency between predicted and reference values. The bias is defined as the difference between the predicted and reference values, and the 95% confidence interval of the bias is calculated. If 0 is within the confidence interval, the bias is acceptable, and the consistency is considered adequate. Otherwise, the bias is unacceptable, and the consistency is deemed insufficient.

## 3. Results and Discussion

### 3.1. Evaluation Metrics for Algorithm Performance

The YOLO v11m-OBB left-and-right ear root recognition model employs three evaluation metrics: Precision (Equation (4)), Recall (Equation (5)), and Mean Average Precision (*mAP*, calculated through Equations (6)–(7)).

The formula for precision (*P*) is as follows:

(4) $$P=\frac{TP}{TP+FP}$$

The formula for recall (*R*) is as follows:

(5) $$R=\frac{TP}{TP+FN}$$

where *TP*, *FP*, *TN*, and *FN* represent the number of true positives, false positives, true negatives, and false negatives, respectively.

The mean average precision (*mAP*) is the average of the average precision (*AP*) for different classes. The formula is as follows:

(6) $$AP=\int_{0}^{1}P(R)dr$$

(7) $$mAP=\frac{1}{c}\sum_{i=1}^{c}{AP}_{i}$$

where *c* represents the number of object classes, *AP* is the average precision for a single class at different recall rates, *P* is precision, and *R* is recall.

The accuracy of left and right ear root pairing is used as the evaluation metric for the ear root pairing algorithm, as shown in Equation (8):

(8) $$A=\frac{P_{c}}{P_{t}}$$

where $P_{c}$ is the number of successfully paired left and right ear roots, and $P_{t}$ is the total number of left and right ear root pairs.

For evaluating the correlation and consistency between the body temperature detection results and the reference values, the Pearson correlation coefficient and bias are used as the evaluation metrics, as shown in Equations (9) and (10):

(9) $$r=\frac{\sum_{i=1}^{n}\left(x_{i}-\bar{x})(y_{i}-\bar{y}\right)}{\sqrt{\sum_{i=1}^{n}{\left(x_{i}-\bar{x}\right)}^{2}}\sqrt{\sum_{i=1}^{n}{\left(y_{i}-\bar{y}\right)}^{2}}}$$

where *n* is the sample size, $y_{i}$ is the reference temperature value, $\bar{y}$ is the mean of reference temperature values, $x_{i}$ is the predicted temperature value, and $\bar{x}$ is the mean of predicted temperature values.

(10) $$Bias=x_{i}-y_{i}$$

### 3.2. Ear Root Prediction Performance

**Figure 7:**
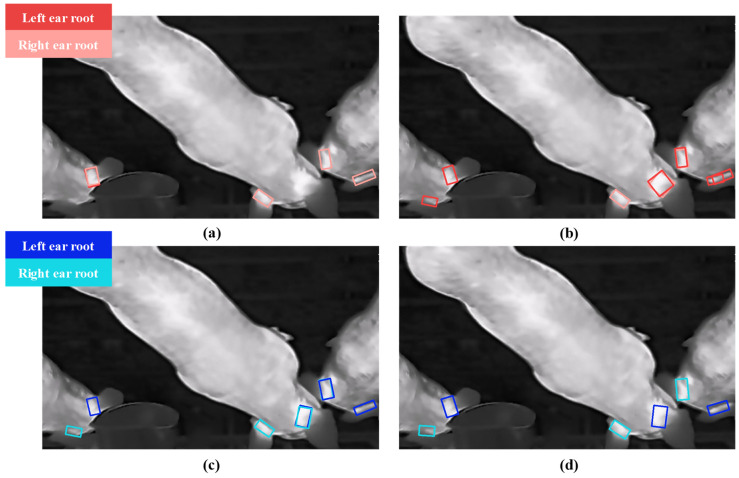
Comparison of ear root detection model results. (**a**) Result of YOLOv8 nano-OBB; (**b**) result of YOLOv8 medium-OBB; (**c**) result of YOLOv11 nano-OBB; (**d**) result of YOLOv11 medium-OBB.

To evaluate the prediction performance of the YOLO model for left and right ear root regions, this study compared several mainstream oriented bounding box (OBB) object detection models, including YOLO v8nano-OBB, YOLO v8medium-OBB, YOLO v11nano-OBB, and YOLO v11medium-OBB. The experimental results are presented in Figure 7 and Table 2.

The YOLO v11medium-OBB algorithm demonstrated the best performance, achieving the highest precision (*P*), recall (*R*), and mean average precision (*mAP*) metrics, with values of 98.7%, 98.4%, and 98.7%, respectively.

**Figure 8:**
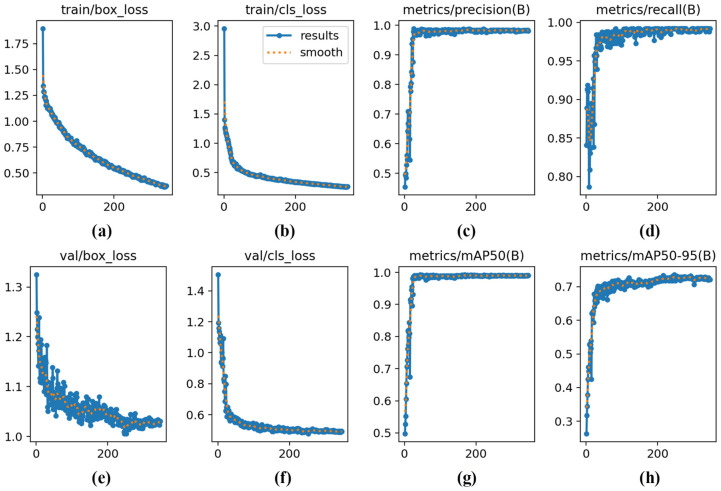
Results of training and validation sets. (**a**) Training—bounding box loss; (**b**) training—classification loss; (**c**) validation—precision; (**d**) validation—recall; (**e**) validation—bounding box loss; (**f**) validation—classification loss; (**g**) validation—mean average precision at 50%; (**h**) validation—mean average precision at 50–95%.

A total of 300 test images were used, containing 749 ear root samples, including 377 left ear samples and 372 right ear samples. In the classification results of the left and right ear root regions, only three false negative samples appeared, including one case of the right ear root and two cases of the left ear root. Additionally, there was one false positive sample, where the background was mistakenly recognized as the left ear root. In addition, the experimental results of YOLO v11m-OBB in the training and validation phases are presented in Figure 8. The results demonstrate that the model performs well in terms of loss value, precision, recall, and mean average precision.

The experimental results demonstrate that YOLO v11 medium-OBB can achieve precise detection of the left and right ear root areas, laying an accurate foundation for subsequent ear root area pairing and individual pig body temperature extraction.

### 3.3. Left and Right Ear Root Pairing Performance

**Figure 9:**
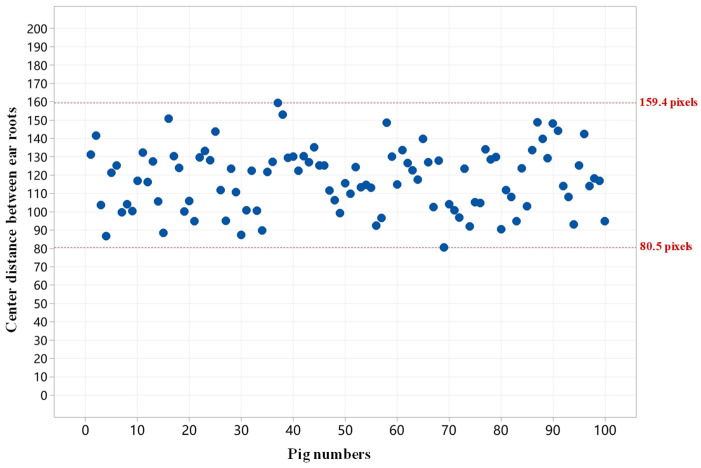
Center distance distribution between left and right ear root bounding boxes.

The pairing of the left and right ear roots is the foundation for extracting the body temperature of individual pigs. The paired ear root regions are the regions of interest for body temperature extraction. During rough pairing, the center distances between the predicted left and right ear root bounding boxes of 100 pigs in thermal images were counted, as shown in Figure 9. It shows that the center distances between the predicted left and right ear root bounding boxes are within 80 to 160 pixels. As a result, this range was used as the criterion for rough matching. To further test the performance of the left and right ear root pairing, the performances of both rough and precise pairing algorithms were evaluated.

A total of 749 left and right ear test samples were used, with 377 left ear samples and 372 right ear samples, resulting in 371 pairs of predicted ear root bounding boxes. Due to three missed detections in the YOLO v11m-OBB ear root predictions, two pairs of ear root predicted bounding boxes were excluded from the pairing.

The performance of 369 pairs of left and right ear root regions was tested through stepwise validation of the rough and precise pairing algorithms. The experimental results are shown in Table 3. The results indicate that after rough pairing, 313 pairs were correctly matched, with an accuracy of 84.8%, and 56 pairs were incorrectly matched, with an error rate of 15.2%. The errors were primarily due to differences in the center distance between the left and right ear roots of individual pigs, as well as the proximity of pigs’ ears when they gathered near a drinking trough, leading to high error rates in the rough pairing algorithm based on the center distance clustering of predicted bounding boxes. Nevertheless, the rough pairing results can serve as the basis for precise pairing. Precise pairing is based on the results of rough pairing, utilizing the angular relationship of the predicted bounding boxes for left and right ear root areas in the polar coordinate system to achieve identification of left and right ear root categories. If the categories are consistent with the YOLO v11m-OBB ear root prediction results, the rough pair is considered correct. Otherwise, the pairing is considered incorrect and is re-paired.

The experimental results demonstrate that the precise pairing algorithm reduced the 56 incorrect pairs identified in the rough pairing stage to just five, correcting 51 errors and achieving an optimization rate of 91.1%. The number of correct pairs increased from 313 in the rough pairing stage to 364 in the precise pairing stage, with accuracy improving from 84.8% to 98.1%. These results highlight that the precise pairing algorithm can significantly enhance the accuracy of matching the left and right ear root predicted bounding boxes.

**Figure 10:**
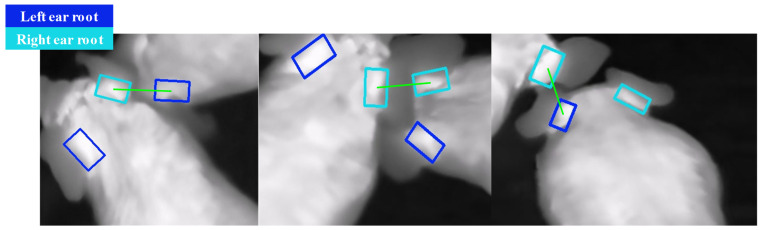
Typical left and right ear root pairing errors.

As shown in Figure 10, the errors in pairing primarily occurred when pigs were positioned at the edges of the image or were in motion, particularly when their heads collided, causing misalignments in the direction of the ear roots and not conforming to the typical characteristics of the left and right ear directions of the pigs.

To further analyze the impact of pig quantity in thermal images on algorithm performance, the images were categorized into single-pig and multi-pig types. A total of 233 single-pig images were used, containing 233 pairs of ear roots, while 67 multi-pig images were used, containing 138 pairs of ear roots. Two pairs of ear roots from the multi-pig images were excluded from the pairing algorithm due to errors in the YOLO v11m-OBB ear root predictions. The performance of the pairing algorithm was evaluated on both image sets, with the results shown in Table 3. For single-pig images, the pairing accuracy during the rough pairing stage reached 100%. In contrast, for multi-pig images, the rough pairing accuracy was only 58%. After applying the TEPA precise pairing algorithm, the accuracy increased to 94.9%. These results indicate that for single-pig images, the rough pairing algorithm accurately measured the center distance between the left and right ear roots within the range of 80 to 160 pixels. However, in multi-pig images, factors such as pig aggregation and the proximity of ears significantly affected the performance of the rough pairing algorithm. The precise pairing algorithm, which incorporates the YOLO v11m-OBB ear root predictions and the angular relationships of the predicted ear root bounding boxes in polar coordinates, effectively corrected the rough pairing errors, thereby improving performance in group-rearing environments.

Using the above method, for single-pig images, the pairing accuracy was 100%, while for multi-pig images, it was 94.9%. Overall, the pairing accuracy was 98.1%, laying a solid foundation for accurately extracting the regions of interest for individual pig body temperature based on left and right ear roots.

### 3.4. Body Temperature Detection Accuracy

Based on the paired ear root regions, the maximum temperature value from both ear root regions is used as the body temperature of the pig. Previous studies have demonstrated the correlation between ear root temperature and the true body temperature of pigs [34,35,36]. Therefore, this study only experimentally verifies the correlation and consistency between the maximum temperature values extracted from the predicted bounding boxes of the bilateral ear root areas, derived from predictions and pairing, and the maximum temperature values obtained manually from the bilateral ear regions. Predicted and reference values of temperature at bilateral ear root areas of 100 pigs were counted. The maximum temperature values extracted from the predicted bounding boxes of individual pigs’ left ear root, right ear root, and the combined bilateral ear root areas were considered as three sets of pig body temperature prediction values. The maximum temperature values manually extracted from the central line area of the ear root in thermal images were used as the reference values for pig body temperature.

To test the correlation between predicted and reference values, correlation analyses were conducted on three sets of predicted values against the reference values. The results showed that the *r* between the predicted value and the reference value in the left ear root was 0.915, the *r* in the right ear root was 0.964, and that in paired ear roots was 0.989. This indicates that when predicting the body temperature of pigs based on a single ear root (either the left or the right), the Pearson correlation coefficient (*r*) between the predicted value and the reference in a single ear root is lower than that in paired ear roots. The temperature prediction values obtained from the right ear are superior to those from the left ear. In comparison, the correlation between the temperature prediction values based on both ears and the reference values is significantly better than those of a single ear.

**Figure 11:**
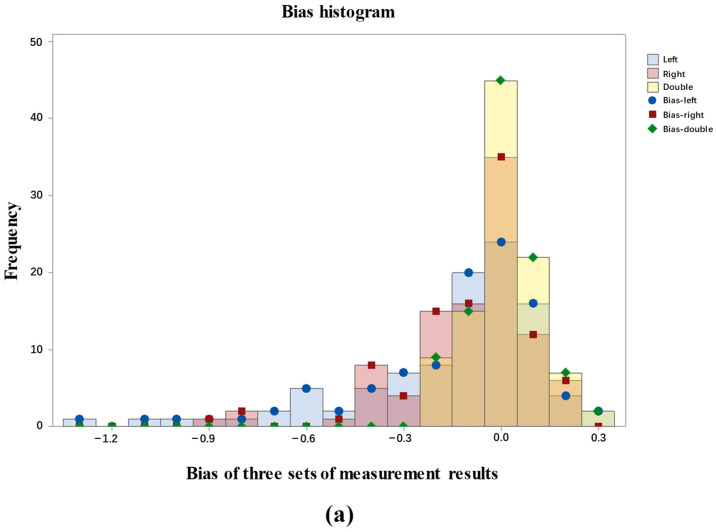

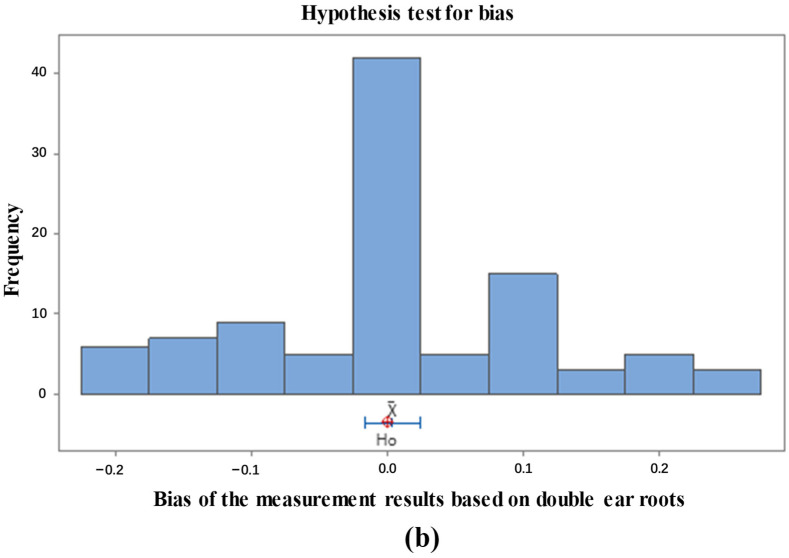
Statistical results of body temperature detection. (**a**) Bias histogram; (**b**) hypothesis test for bias.

A consistency analysis was conducted based on bias values between the predicted body temperature values derived from bilateral ear root areas and the reference values. The results are shown in Figure 11. The results indicate that the mean bias is 0.014 °C and the standard deviation is 0.103 °C for measurement results based on double ear roots. A hypothesis test for a bias equal to zero was performed, with zero falling within the 95% confidence interval of the bias, and a *p*-value of 0.701, which is greater than 0.05. Thus, the bias is considered acceptable. Consequently, the hypothesis test results demonstrate that the body temperature prediction values extracted from both ear root areas are significantly consistent with the reference values.

## 4. Conclusions

The thermal image acquisition system captured thermal images of pigs near the drinking trough area from a top-down view to ensure that the ear roots of the pigs are fully exposed without obstruction while pigs maintain a standing posture at the drinking station. This is very helpful for obtaining the temperature value of the ear root.

The rough pairing algorithm, based on the center distance clustering of the left and right ear root predicted bounding boxes, achieves 100% accuracy in single-pig images. However, due to factors such as ear proximity in multi-pig group-rearing environments, the accuracy in multi-pig images was only 58%. The precise pairing algorithm, based on the polar coordinate system, significantly improves pairing accuracy by utilizing the predicted ear root categories and the angular relationships of the predicted bounding boxes. The two-stage rough and precise left–right ear pairing algorithm effectively defines the regions of interest for body temperature extraction based on both ears, ensuring that body temperature is extracted only from the ear root regions.

The correlation between the body temperature prediction values based on bilateral ear root areas and the reference values is superior to that based on a single ear. The Pearson correlation coefficient (*r*) between the body temperature prediction values based on bilateral ear root areas and the reference values is 0.989. The mean bias between the body temperature prediction values based on bilateral ear root areas and the reference values is 0.014 °C, with a standard deviation of 0.103 °C. The body temperature prediction values based on bilateral ear root areas are both correlated with and consistent with the reference values.

To address the issue of mismatching between left and right ear root areas, further research could integrate multi-spectral image fusion and object tracking to explore the facial features, body posture characteristics, and behavioral patterns of pigs, thereby achieving precise identification of individual pigs [37,38,39]. In addition, considering rectal temperature as the gold standard for pig body temperature, further design of the body temperature correction model is necessary, based on the rectal temperature of pigs measured manually by a thermometer or implantable sensors. External environmental factors such as temperature and humidity should also be considered to establish a pig body temperature prediction model and explore the impact of environmental factors on pig body temperature.

## Figures and Tables

**Table 1:** Experimental platform.

Component Name	Configuration
CPU	Intel(R) Core (TM) i5-12400f
Memory	16 GB
GPU	NVIDIA GeForce RTX 3060 12 GB
GPU Acceleration	CUDA11.8, CUDNN8.9.2
Operating System	Windows 10 Professional (64-bit)
Algorithm Environment	Python 3.9.20, Torch 2.2.1, Ultralytics 8.3.25

Note: All experiments were conducted on a single computer.

**Table 2:** Comparison of mainstream OBB model experimental results.

Model	*P* (%)	*R* (%)	*mAP* (%)
YOLO v8nano-OBB	91.1	91.1	93.4
YOLO v8medium-OBB	94.7	93.1	96.3
YOLO v11nano-OBB	95.5	95.3	97.6
YOLO v11 medium-OBB	**98.7**	**98.4**	**98.7**

Note: The bold part indicates the best value.

**Table 3:** Left and right ear root pairing results.

Statistic	YOLO TEPA-OBB	Ear Root Pairing Results (Pairs)		Total Ear Roots (Pairs)	Pairing Accuracy (%)
		Incorrect	Correct		
All Test Images	Rough Pairing	56	313	371	84.8%
	Precise Pairing	5	364		98.1%
Single-Pig Images	Rough Pairing	0	233	233	100%
	Precise Pairing	0	0	0	0%
Multi-Pig Images	Rough Pairing	56	80	138	58%
	Precise Pairing	5	131		94.9%

## Data Availability

All data included in this study are available upon request by contacting the corresponding author.

## References

[1] Tzanidakis C., Simitzis P., Arvanitis K., Panagakis P. (2021). An Overview of the Current Trends in Precision Pig Farming Technologies. Livest. Sci..

[2] Wang M., Larsen M.L.V., Liu D., Winters J.F.M., Rault J.-L., Norton T. (2022). Towards Re-Identification for Long-Term Tracking of Group Housed Pigs. Biosyst. Eng..

[3] Wang S., Jiang H., Qiao Y., Jiang S., Lin H., Sun Q. (2022). The Research Progress of Vision-Based Artificial Intelligence in Smart Pig Farming. Sensors.

[4] Godyń D., Herbut P. (2018). Applications of Continuous Body Temperature Measurements in Pigs—A Review. Ann. Wars. Univ. Life Sci.-SGGW-Anim. Sci..

[5] Albernaz-Gonçalves R., Olmos G., Hötzel M.J. (2021). My Pigs Are Ok, Why Change?—Animal Welfare Accounts of Pig Farmers. Animal.

[6] Yang Q., Hui X., Huang Y., Chen M., Huang S., Xiao D. (2024). A Long-Term Video Tracking Method for Group-Housed Pigs. Animals.

[7] Zhang Z., Zhang H., Liu T. (2019). Study on Body Temperature Detection of Pig Based on Infrared Technology: A Review. Artif. Intell. Agric..

[8] Jia G., Li W., Meng J., Tan H., Feng Y. (2020). Non-Contact Evaluation of Pigs’ Body Temperature Incorporating Environmental Factors. Sensors.

[9] Zhao H. (2019). Pig Body Temperature Detection and Key Temperature Measurement Part Recognition. Master’s Thesis.

[10] Pandey S., Kalwa U., Kong T., Guo B., Gauger P.C., Peters D.J., Yoon K.-J. (2021). Behavioral Monitoring Tool for Pig Farmers: Ear Tag Sensors, Machine Intelligence, and Technology Adoption Roadmap. Animals.

[11] Hentzen M., Hovden D., Jansen M., van Essen G. (2012). Design and Validation of a Wireless Temperature Measurement System for Laboratory and Farm Animals. Proc. Meas. Behav..

[12] Zhang Z. (2015). Detected Onset of Estrus and Behavior in the Different Varieties of Gilts Using the New Electronic Chip. Master’s Thesis.

[13] McManus C., Tanure C.B., Peripolli V., Seixas L., Fischer V., Gabbi A.M., Menegassi S.R.O., Stumpf M.T., Kolling G.J., Dias E. (2016). Infrared Thermography in Animal Production: An Overview. Comput. Electron. Agric..

[14] Zhang B., Xiao D., Liu J., Huang S., Huang Y., Lin T. (2024). Pig Eye Area Temperature Extraction Algorithm Based on Registered Images. Comput. Electron. Agric..

[15] Zheng S., Zhou C., Jiang X., Huang J., Xu D. (2022). Progress on Infrared Imaging Technology in Animal Production: A Review. Sensors.

[16] Pacheco V.M., Sousa R.V., Sardinha E.J.S., Rodrigues A.V.S., Brown-Brandl T.M., Martello L.S. (2022). Deep Learning-Based Model Classifies Thermal Conditions in Dairy Cows Using Infrared Thermography. Biosyst. Eng..

[17] Gao L., Duan G., Yin G., Zou F., Ynag L., Yang F. (2010). Trial Results of Infrared Thermometer in the Application of Ante-mortem Body Temperature Screening in the Pig Slaughterhouse. China Anim. Husb. Vet. Med..

[18] Soerensen D.D., Pedersen L.J. (2015). Infrared Skin Temperature Measurements for Monitoring Health in Pigs: A Review. Acta Vet. Scand..

[19] Zhong Z. (2022). A Novel Visible and Infrared Image Fusion Method Based on Convolutional Neural Network for Pig-Body Feature Detection. Multimed. Tools Appl..

[20] Yamsakul P., Yano T., Na Lampang K., Khamkong M., Srikitjakarn L. (2022). Infrared Temperature Sensor for Use Among Sow Herds. Vet. Integr. Sci..

[21] Mazur-Milecka M., Ruminski J. (2021). Deep Learning Based Thermal Image Segmentation for Laboratory Animals Tracking. Quant. InfraRed Thermogr. J..

[22] Zhang Z., Wang H., Liu T., Wang Y., Zhang H., Yuan F., Yang X., Xu S., Meng Y. (2021). Accurate Detection Method of Pig’s Temperature Based on Non-point Source Thermal Infrared Image. CAAI Trans. Intell. Technol..

[23] Jiao L., Dong D., Zhao X., Han P. (2016). Compensation Method for the Influence of Angle of View on Animal Temperature Measurement Using Thermal Imaging Camera Combined with Depth Image. J. Therm. Biol..

[24] Feng Y., Kang X., Wang Y., Li M., Liu G. (2021). Detecting Method of Surface Temperature of Pig Ear Root Based on Thermal Infrared Video. Trans. Chin. Soc. Agric. Mach..

[25] Xiao D., Lin S., Liu Q., Huang Y., Zeng R., Chen L. (2021). Automatic Ear Temperature Extraction Algorithm for Live Pigs Based on Infrared Thermography. Trans. Chin. Soc. Agric. Mach..

[26] Xie Q., Wu M., Bao J., Zheng P., Liu W., Liu X., Yu H. (2023). A Deep Learning-Based Detection Method for Pig Body Temperature Using Infrared Thermography. Comput. Electron. Agric..

[27] Liu X., Zeng X., Li T., Liu G., Ding X., Mi Y. (2023). Automatic Detection Method of Body Temperature in Herd of Pigs Based on Improved YOLO v7. Trans. Chin. Soc. Agric. Mach..

[28] Tabuaciri P., Bunter K., Graser H.-U. (2016). Thermal Imaging as a Potential Tool for Identifying Piglets at Risk. AGBU Pig Genet. Workshop.

[29] Tian H., Hua J., Zhang S., Liu L. (2023). Research on the measurement of sow body temperature based on infrared thermography and linear regression fitting. J. Intell. Agric. Mech..

[30] Brown-Brandl T.M., Hayes M.D., Rohrer G.A., Eigenberg R.A. (2023). Thermal Comfort Evaluation of Three Genetic Lines of Nursery Pigs Using Thermal Images. Biosyst. Eng..

[31] Lu M., He J., Chen C., Okinda C., Shen M., Liu L., Yao W., Norton T., Berckmans D. (2018). An Automatic Ear Base Temperature Extraction Method for Top View Piglet Thermal Image. Comput. Electron. Agric..

[32] Mu Y., Hu J., Wang H., Li S., Zhu H., Luo L., Wei J., Ni L., Chao H., Hu T. (2024). Research on the Behavior Recognition of Beef Cattle Based on the Improved Lightweight CBR-YOLO Model Based on YOLOv8 in Multi-Scene Weather. Animals.

[33] Guarnido-Lopez P., Ramirez-Agudelo J.-F., Denimal E., Benaouda M. (2024). Programming and Setting Up the Object Detection Algorithm YOLO to Determine Feeding Activities of Beef Cattle: A Comparison between YOLOv8m and YOLOv10m. Animals.

[34] Hu F. (2023). Research on Identification Method of Group-Housed Nursery Pigs with Abnormal Body Temperature Using Infrared Thermography. Master’s Thesis.

[35] Huang Y. (2024). Analysis of Pig Activity Level and Body Temperature Variation Based on Ear Tag Data. Comput. Electron. Agric..

[36] Wang X., Hu F., Yang R., Wang K. (2023). An Infrared Temperature Correction Method for the Skin Temperature of Pigs in Infrared Images. Agriculture.

[37] Tu S., Ou H., Mao L., Du J., Cao Y., Chen W. (2024). Behavior Tracking and Analyses of Group-Housed Pigs Based on Improved ByteTrack. Animals.

[38] Xiao D., Lin S., Liu Y., Yang Q., Wu H. (2022). Group-Housed Pigs and Their Body Parts Detection with Cascade Faster R-CNN. Int. J. Agric. Biol. Eng..

[39] Jiang S., Zhang G., Shen Z., Zhong P., Tan J., Liu J. (2024). Pig Weight Estimation Method Based on a Framework Combining Mask R-CNN and Ensemble Regression Model. Animals.

